# Reproducibility of Resting State Connectivity in Patients with Stable Multiple Sclerosis

**DOI:** 10.1371/journal.pone.0152158

**Published:** 2016-03-23

**Authors:** Daniela Pinter, Christian Beckmann, Marisa Koini, Eva Pirker, Nicola Filippini, Alexander Pichler, Siegrid Fuchs, Franz Fazekas, Christian Enzinger

**Affiliations:** 1 Department of Neurology, Medical University of Graz, Graz, Austria; 2 Donders Institute, Cognitive Neuroscience Department and Centre for Cognitive Neuroimaging, Radboud University Nijmegen, Nijmegen, The Netherlands; 3 The Oxford Centre for Functional MRI of the Brain, Nuffield Department of Clinical Neurosciences, University of Oxford, Oxford, United Kingdom; 4 Division of Neuroradiology, Department of Radiology, Medical University of Graz, Graz, Austria; Institute of Psychology, Chinese Academy of Sciences, CHINA

## Abstract

Given increasing efforts to use resting-state fMRI (rfMRI) as a biomarker of disease progression in multiple sclerosis (MS) we here explored the reproducibility of longitudinal rfMRI over three months in patients with clinically and radiologically stable MS. To pursue this aim, two approaches were applied in nine rfMRI networks: First, the intraclass correlation coefficient (ICC 3,1) was assessed for the mean functional connectivity maps across the entire network and a region of interest (ROI). Second, the ratio of overlap between *Z*-thresholded connectivity maps for each network was assessed. We quantified between-session functional reproducibility of rfMRI for 20 patients with stable MS and 14 healthy controls (HC). Nine rfMRI networks (RSNs) were examined at baseline and after 3 months of follow-up: three visual RSNs, the default-mode network, sensorimotor-, auditory-, executive control, and the left and right fronto-parietal RSN. ROI analyses were constrained to thresholded overlap masks for each individual (Z>0) at baseline and follow-up.In both stable MS and HC mean functional connectivity across the entire network did not reach acceptable ICCs for several networks (ICC<0.40) but we found a high reproducibility of ROI ICCs and of the ratio of overlap. ROI ICCs of all nine networks were between 0.98 and 0.99 for HC and ranged from 0.88 to 0.99 in patients with MS, respectively. The ratio of overlap for all networks was similar for both groups, ranging from 0.60 to 0.75.Our findings attest to a high reproducibility of rfMRI networks not only in HC but also in patients with stable MS when applying ROI analysis. This supports the utility of rfMRI to monitor functional changes related to disease progression or therapeutic interventions in MS.

## Introduction

Multiple sclerosis (MS) is an inflammatory, neurodegenerative disease [1–3] and the major cause for non-traumatic disability in young adults [4]. Physical and cognitive deficits of MS have been related not only to structural damage but also to functional imbalance in and between brain networks [5]. Therefore, the study of functional changes of the brain by fMRI holds great promise to better understand the pathophysiologic mechanisms of the disease and their modification by therapeutic interventions [6]. Given recent increasing propositions to use resting-state fMRI (rfMRI) as a biomarker of disease progression and to monitor and/or predict motor and cognitive function in MS [7–9], we here explored the reproducibility of rfMRI over three months in patients with stable MS and compared findings to healthy controls.

RfMRI allows the investigation of changes within and across multiple functional networks without bias of task performance, adherence or subject effort and is increasingly used in patient cohorts [10,11]. Independent component analysis (ICA) has emerged as a powerful tool for exploring rfMRI data in both healthy and brain-diseased populations [12].

A high test-retest reproducibility of fMRI data is a pre-requisite for their application in clinical practice and clinical populations [13–15]. Comparing group activation maps is not ideal for establishing reproducibility of fMRI signals [16], as the step of statistical thresholding of images can exaggerate very small differences between maps [17]. Hence, computing intraclass correlation coefficients (ICCs) is frequently recommended to assess fMRI reproducibility [18,19].

Recent studies tested reproducibility of fMRI activation in patients with MS using task-related fMRI for motor and cognitive functions [20,21]. However, advantages of rfMRI (e.g. no limitation due to a specific task, no ceiling and floor effects related to cognitive or behavioral constraints) lead to an increasing use of rfMRI to explore MS-related changes of brain integrity in cross sectional and longitudinal settings [10]. Although empirical evidence suggest high levels of converging findings, so far no study explicitly investigated reproducibility of longitudinal rfMRI in MS. Therefore, we quantified and compared between-session reproducibility of rfMRI derived network structure for patients with MS and healthy controls in terms of ICC values [18,19] and the ratio of overlap [22,23].

## Materials and Methods

### 1.1. Participants

24 patients with MS and 15 age- and sex matched healthy controls (HC) underwent comprehensive 3T MRI (T1- and FLAIR-weighted imaging, rfMRI) at baseline (BL) and after three months of follow-up (FU). The study was approved by the ethics committee of the Medical University of Graz. All participants gave written informed consent.

Patients were selected from the MS outpatient clinic of the Department of Neurology, Medical University of Graz if they had a clinically and radiologically stable disease and were on continuous disease-modifying treatment (glatiramer-acetate, β-interferons or natalizumab). Only patients with a diagnosis of relapsing-remitting (RR) clinically-definite MS were considered [24]. Patients had to have no relapse within the previous two months, had not received corticosteroids eight weeks prior to inclusion, and had no history of serious psychiatric illness (e.g. depression) or other neurologic disorders. All patients underwent additional clinical and neuropsychological testing to control for disease activity.

MRI data of four patients and one healthy control finally had to be excluded (four due to scanner or movement artefacts, one patient did not attend the follow-up assessment), resulting in a final sample of 14 healthy controls and 20 patients (see Table 1 for characteristics).

**Table 1:** Demographics and morphological MRI characteristics of healthy controls (HC) and patients with multiple sclerosis (MS).

	HC	MS	p
N	14	20	
Sex, male, n (%)	6 (42)	5 (25)	.27
Age (years)	29.1 (10.3)	33.1 (9.3)	.24
Education (years)	15.4 (3.7)	13.7 (3.3)	.17
NBV (cm^3^)	1584.5 (83.3)	1511.1 (79.5)	.01
PBVC	-0.2 (0.4)	-0.2 (0.7)	.91

NBV = normalized brain volume. PBVC = percent brain volume change. The Chi-Square test was applied to compare the groups according to sex. Unpaired t-tests were used to compare the groups according to age, education, NBV and PBVC.

### 1.2. Clinical and Neuropsychological Assessment

In patients with MS disability was measured using the Expanded Disability Status Scale (EDSS) [25] both at baseline and a 3-months follow-up. To capture possible changes in the cognitive subdomains known to be most sensitive to change in MS, we assessed the subtest for episodic memory of the Wechsler memory scale (WMS) [26], and two subtests of the Brief Repeatable Battery of Neuropsychological Tests (BRB-N) [27], namely the Symbol Digit Modalities Test (SDMT; information processing speed, sustained attention, and concentration) and Paced Auditory Serial Addition Test (3-second version; PASAT; sustained attention and concentration) at BL and FU, using parallel test forms if available.

### 1.3. MRI

MRI was performed on a 3 Tesla TimTrio scanner (Siemens Healthcare, Erlangen, Germany) using a 12-channel head coil. High-resolution structural 3D images were acquired by means of a T1-weighted MPRAGE sequence with 1 mm isotropic resolution (TR = 1900 ms, TE = 2.19 ms, 176 slices). A fluid-attenuated inversion recovery (FLAIR) sequence with 1x1x3 mm^3^ resolution served for the assessment of the hyperintense T2-lesion load in the patients (T2-LL; TR = 9000 ms; TE = 69 ms, 44 slices). RfMRI data were acquired with a 3x3x3.75 mm resolution (Sequence = 2D echo planar imaging (EPI), TR = 3000 ms; TE = 30; 150 volumes, field of view = 192 x 192 mm^2^, Matrix = 64 x 64, 36 slices, slice thickness = 3 mm, acquisition time = 7.5 minutes). Patients were asked to close their eyes during the rfMRI sequence. The total imaging time was approximately 20 minutes.

### 1.4. Image Analysis

#### 1.4.1. Structural Analyses

Normalized Brain Volume (NBV; in cm^3^) was estimated from the T1-weighted MPRAGE images using SIENAX (Structural Image Evaluation, using Normalisation, Single-Time-Point Estimation v 2.6), part of the FMRIB Software Library (FSL).

Percent brain volume change (PBVC) was assessed using SIENA (Structural Image Evaluation, using Normalisation; Two-Time-Point Estimation v 2.6). T2-lesion load (T2-LL) was assessed by a semi-automated region growing algorithm [28] subsequent to lesion identification by an experienced rater.

#### 1.4.2. Functional Analyses—rfMRI

In the first step, individual resting state data were preprocessed using FEAT (FMRIB's Expert Analysis Tool, v 6.0, part of FSL v 5.0.4 [29]. Individual pre-statistical processing included: motion correction using MCFLIRT, brain extraction, spatial smoothing using a Gaussian kernel of FWHM (full width at half maximum) of 6 mm [30], high pass temporal filtering using a cut-off of 150 s (0.007 Hz), linear registration to main structural image (BBR) and nonlinear registration warp resolution of 10 mm. High-resolution T1 scans served as targets for image registration. Next, Independent Component Analysis (ICA) was used for rfMRI data exploration (FSL-MELODIC, v 3.12), denoising the data and filtering out components based on high vs. low frequency content (fsl_regfilt command line tool). The resulting denoised functional images were resampled to standard space (MNI152 template 2 mm). Dual-regression analyses against ten resting-state templates from 36 healthy controls [31,32] on the denoised, registered functional images of each subject was performed in order to obtain individual spatial maps of the networks.

**Fig 1:**
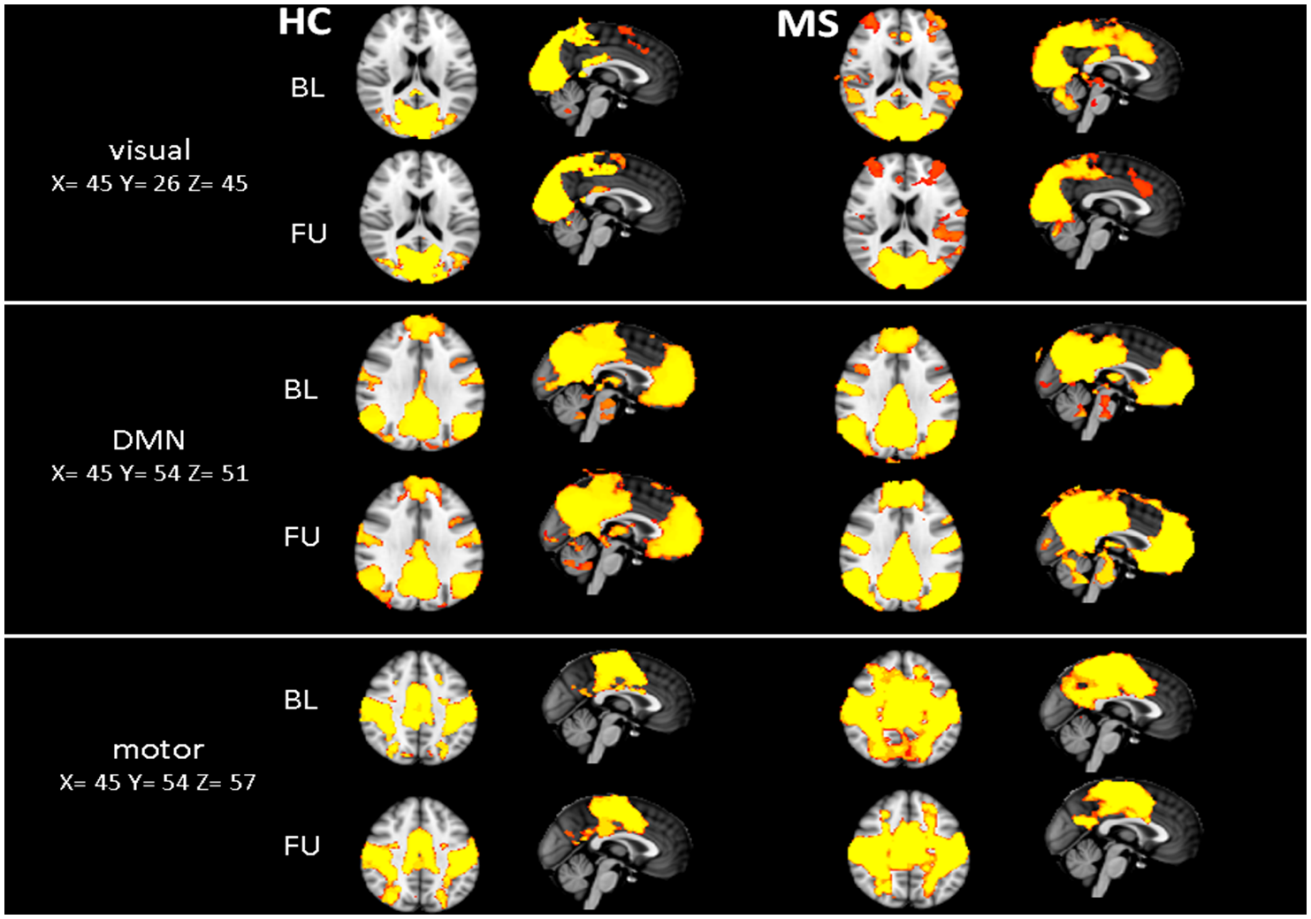
Group functional connectivity maps. Fig 1 illustrates the visual network (visual 00), the default-mode network (DMN) and sensorimotor network (motor) at baseline (BL) and follow-up (FU) for both groups. Tfce corrected p-values > 0.99. HC = Healthy controls, MS = patients with MS.

Group functional connectivity maps for BL and FU were computed for both groups and assessed for statistical significance (using FSL Randomise; see Fig 1). Furthermore, for each subject the longitudinal change of resting-state connectivity was individually computed and the difference maps were used to assess pre-post differences of rfMRI-connectivity networks at group level (using FSL Randomise).

#### 1.4.3. Intraclass Correlation Coefficient (ICC)

The ICC assesses reproducibility by comparing the between-subject variance to total variance and it is therefore higher when within-subject variance is low and between-subject variance is high. The ICC for each network was assessed for the mean functional connectivity maps across the entire network of the group (EN) and a region of interest (ROI).

Regions of interest (ROI) were obtained by overlap masks for each individual (Z>0) at BL and FU and the ICC [19] were calculated as:

ICC (3,1)=(BMS−EMS)÷(BMS+(k−1)EMS)

where BMS is the between subject mean square error, EMS the error mean square and k the number of comparisons. This formula estimates the correlation of the subject signal intensities between sessions, modeled by a two-way ANOVA, with random subject effects and fixed session effects. An ICC value approaching 1 indicates high reproducibility, while a value close to 0 indicates very low reproducibility.

#### 1.4.4. Ratio of overlapping connectivity (R12)

We assessed the ratio of overlapping functional connectivity by using the Dice coefficient [23]:

R12=2×V−overlap÷(V1+V2)

Where V1 and V2 denote the number of suprathreshold voxels (Z>0) in the Z-volume at BL and FU, respectively, and V-overlap stands for the number of suprathreshold voxels in both Z-volumes. The R12 can range from 0 (no overlap) to 1 (perfect overlap) [13,33].

### 1.5. Statistical Analysis

Clinical, morphological and reproducibility scores were analyzed with the Statistical Package of Social Science (IBM SPSS Statistics 23). The level of significance was set at 0.05. As nine rfMRI networks were compared, for reproducibility scores a Bonferroni-adjusted level of significance of 0.0056 was applied. Baseline comparisons between groups and within-group comparisons were done using t-tests.

## Results

### 2.1. Behavioral and Morphological findings

During the 3 months follow-up period patients with MS showed no clinical or morphological (MRI) evidence of disease activity (i.e. new lesions, enlarging lesions or new gadolinium-enhanced lesions), no change of T2-LL, EDSS and cognitive (memory, processing speed, attention) function (see Table 2). Slight improvements in memory performance were observed, most likely representing a learning effect, as no parallel version of the WMS was available. There were no measurable changes in global brain volume over three months for both groups (Table 1).

**Table 2:** Disease activity (T2-LL), motor and cognitive function at baseline (BL) and follow-up (FU) for patients with multiple sclerosis (MS).

	BL	FU	p
T2-LL (cm^3^)	5.5 (4.9)	6.1 (5.5)	.06
EDSS	2.3 (1.2)	2.2 (1.3)	.69
Memory	30.2 (5.4)	32.1 (4.5)	.02
Processing Speed	51.5 (10.4)	53.2 (10.2)	.35
Attention	47.2 (9.9)	48.3 (9.2)	.52

T2-LL = Hyperintense T2-lesion load, EDSS = Expanded Disability Status Scale. Values indicate means +/- 1 standard deviation. The Wilcoxon test was used to compare BL and FU EDSS scores. Paired t-tests were used to compare BL and FU scores of T2-LL, Memory, Processing Speed and Attention in patients with MS.

### 2.2. Identification and Comparison of resting-state networks at baseline and follow-up

Nine rfMRI networks were investigated in both groups: three visual networks (00, 01, 02), the default-mode network (DMN; 03), one sensorimotor network (05), one auditory network (06), one executive control network (07), and a left and right fronto-parietal network (08, 09) (individual networks in line with Smith et al., 2009; available at http://www.fmrib.ox.ac.uk/analysis/brainmap+rsns/). The cerebellar network (04) was not examined as the most inferior slices of the cerebellum had been cut off in some subjects to ensure full coverage of the cortex.

Pre-post comparison, including individual difference maps for each group revealed no significant changes in HC and MS. Fig 1 illustrates exemplarily three of the nine networks (the visual network (00), DMN and sensorimotor network) at BL and FU for both groups separately (all nine networks are illustrated in the supplement S1 Fig).

### 2.3. Reproducibility of resting-state networks assessed by ICC

Entire network (EN) ICC scores of the HC ranged from 0.73 (visual network 01) to 0.00 (auditory and left fronto-parietal network). For patients with MS, EN ICC’s were generally lower, ranging from 0.42 (auditory network) to 0.00 (visual 02 and sensorimotor network; Table 3).

**Table 3:** Intraclass correlation coefficients (ICCs) of separate networks for HC and patients with MS, for mean of the entire network connectivity (EN) and mean of the ROI connectivity. ICC values of task-related fMRI for HC and patients with MS (hand tapping) as reported by Bosnell et al. (2008), are 0.91 and 0.76, respectively.

ICC	HC		MS	
	EN	ROI	EN	ROI
**Visual 00**	0.21	0.99	0.33	0.95
**Visual 01**	0.73	0.99	0.04	0.95
**Visual 02**	0.21	0.99	0.00	0.98
**DMN**	0.28	0.97	0.03	0.88
**Sensorimotor**	0.54	0.98	0.00	0.99
**Auditory**	0.00	0.98	0.42	0.97
**Executive control**	0.11	0.99	0.37	0.99
**Left fronto-parietal**	0.00	0.97	0.18	0.97
**Right fronto-parietal**	0.51	0.97	0.28	0.97

ROI ICCs for the HC across all networks were between 0.97 and 0.99. The highest reproducibility was found for two visual networks and the executive control network. ROI ICCs for patients with MS were between 0.88 and 0.99 across all networks. The highest ROI reproducibility was found for the executive control network. The lowest ROI reproducibility for patients with MS was found in the DMN.

Compared to patients with MS, controls obtained higher EN and ROI ICC scores across all networks, which however were not significant after Bonferroni-adjustment (*p* = 0.0056).

### 2.4. Reproducibility of resting-state networks assessed by overlapping connectivity maps

Mean overlap of functional connectivity across all networks was between 0.61 and 0.75, for HC and between 0.60 and 0.75 in patients with MS. Highest overlap of Z-thresholded connectivity maps (Z>0) was found in the DMN (0.75), for HC and patients with MS. For HC, the lowest R12 reproducibility was observed in the executive control network. For patients with MS, the lowest R12 reproducibility was found in the visual 02 network.

**Fig 2:**
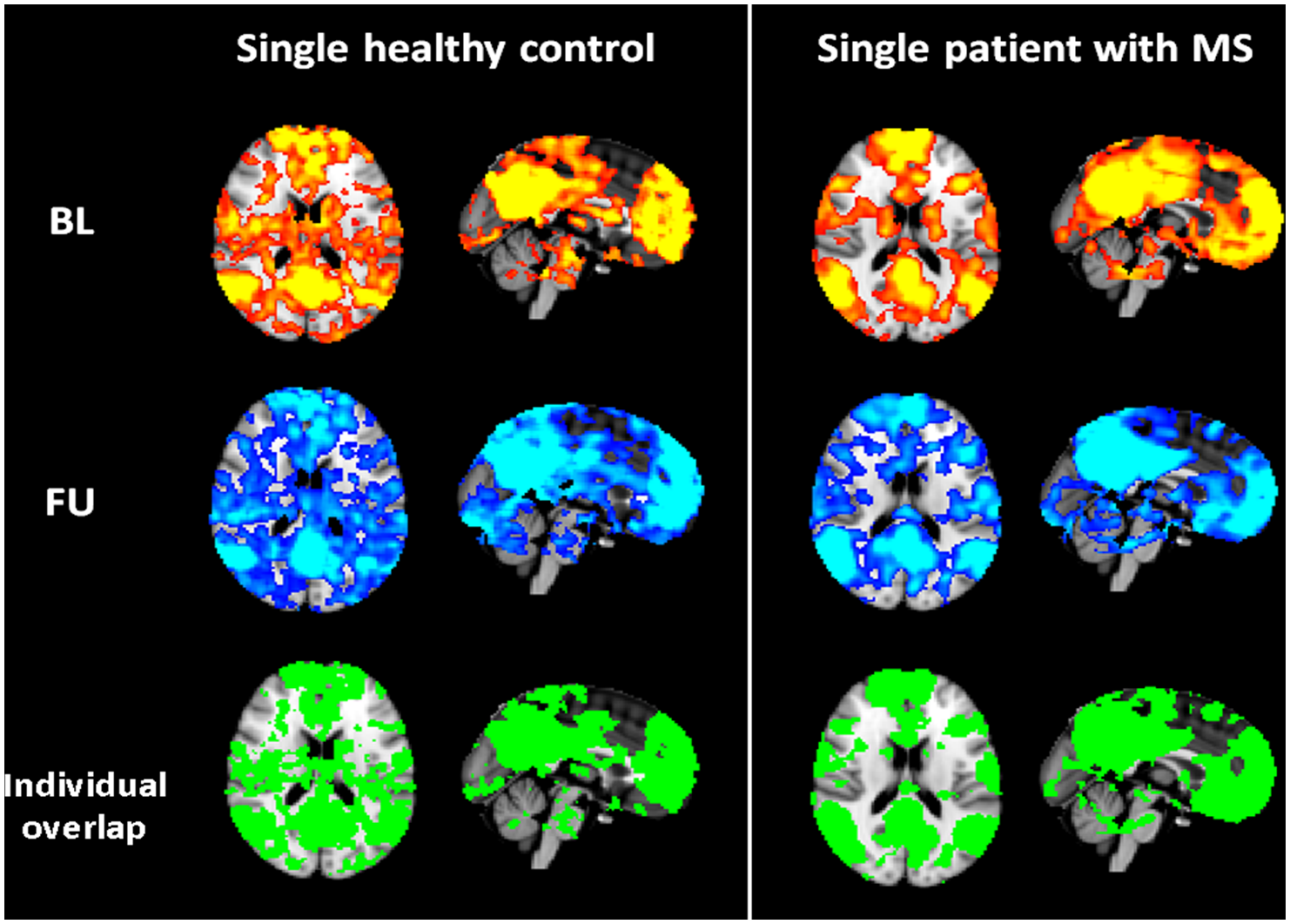
Representative overlapping maps. Representative connectivity maps for baseline (BL), follow-up (FU) and overlap of Z-thresholded (Z>0) connectivity maps for the DMN (X = 45, Y = 54, Z = 45) for a single healthy control and a single patient with MS.

Overlap of Z-thresholded functional connectivity maps (Z>0) did not differ significantly between groups across all nine networks (Table 4, Fig 2).

**Table 4:** Mean overlap (R12) of individual Z-thresholded connectivity maps (Z>0) for each network for HC and MS.

R12	HC	MS	p
**Visual 00**	0.65 (0.06)	0.65 (0.08)	.91
**Visual 01**	0.70 (0.08)	0.69 (0.07)	.81
**Visual 02**	0.62 (0.05)	0.60 (0.09)	.44
**DMN**	0.75 (0.05)	0.75 (0.06)	.89
**Sensorimotor**	0.69 (0.05)	0.68 (0.07)	.77
**Auditory**	0.70 (0.05)	0.65 (0.10)	.10
**Executive control**	0.61 (0.07)	0.65 (0.09)	.27
**Left fronto-parietal**	0.70 (0.04)	0.72 (0.06)	.35
**Right fronto-parietal**	0.71 (0.05)	0.71 (0.09)	.89

## Discussion and Conclusions

This study showed high reproducibility of rfMRI over three months as assessed by ROI ICCs (0.97–0.99 and 0.88–0.99) and ratio of overlap (0.61–0.75 and 0.60–0.75) for HC and patients with stable MS, respectively. ICCs (of entire network and ROI) showed comparable reproducibility in HC and patients with MS and also the ratio of overlap did not differ between groups.

Mean connectivity across the entire network did not reach acceptable ICCs in both groups for several networks (e.g., visual 00, 02, DMN, executive control; ICC<0.40), highlighting the importance of choosing the proper ROI for extraction of signal change [17]. Various approaches to assess ICCs have been explored. Typically, a summary statistic for each subject is obtained for a ROI. This can be the mean or median contrast value within the region, or the value of the contrast at the peak of group activation [18]. The specific ROI frequently confines to an area most likely to be activated due to a task (e.g., sensorimotor cortex for a motor task, or insula for a cognitive task [20,21,34]). Given our data-driven analytic approach of rfMRI, using ICA with dual regression [12,35], we refrained from using anatomically defined ROIs, such as the precuneus (e.g., frequently used as a seed for the default mode network) and instead used the entire network maps at group level and individual overlap masks at BL and FU as shown in Fig 2. The latter proved to provide highly reproducible ICCs. The application of an entire network mask might thus be too over-inclusive to assess rfMRI reproducibility in terms of a single mean score. To assess reproducibility across whole-brain activation, a more sophisticated analytical approach, such as voxel-wise ICC analysis [18] would be more appropriate.

While several studies have investigated reproducibility of task-related fMRI [16,17,36,37], less is known about the reproducibility of rfMRI [13,38,39]. Kristo et al. (2014) compared the reproducibility of task-free fMRI and task motor activity in 16 healthy subjects with a test-retest interval of seven weeks. They found that although both approaches properly identified critical brain areas for motor task performance, rfMRI was less reliable compared to task-related fMRI. Nevertheless, Chou et al. (2012) reported the reproducibility of rfMRI measures to be outstanding and potentially suitable as biomarker for disease progression and treatment effects in clinical trials and individual patients [14]. Recent studies showed moderate to high test-retest reliability of rfMRI in healthy controls [38,40,41]. High reproducibility of longitudinal fMRI has also been reported across multiple sites for task-related and rfMRI [34,42]. However, these previous longitudinal assessments of variability in fMRI have been carried out predominantly using healthy controls [20].

Cross-sectional rfMRI studies in patients with MS suggest that prominent functional changes can be detected in many networks (e.g. default mode or sensorimotor network) and correlate with clinical and/or structural MRI measures [5,43]. Furthermore, the use of functional imaging techniques has highlighted that cortical reorganization might have a role in limiting the clinical consequences of tissue damage [5,44]. The application of rfMRI thus bears great potential to monitor disease progression or the impact of therapeutic intervention. It can be easily added to standard MRI and is not influenced by task-performance. RfMRI could complement structural conventional and quantitative MRI techniques, in order to better understand MS pathophysiology of, for instance, the correlates of cognitive dysfunctioning [45]. However, as Filippi et al. (2013) highlighted, rfMRI still requires a careful standardization of acquisition and analysis protocols, a careful assessment of scanner stability and intraindividual variability over time, and normative values as a reference [5].

In the presented study, we used a short (7.5 minutes) rfMRI-sequence, instructing the patients to close their eyes and explored intraindividual variability over time. We chose to assess two different measures of reproducibility (ICC and R12), that are frequently used and can be easily obtained. Both scores provide complementary information and for the given reasons might be useful as an outcome variable in clinical populations. Although the ICC might be less informative in clinical setting as clinicians most likely interpret fMRI results based on conventional P or Z-thresholding [13], some researchers underlined the poor test–retest reliability of suprathreshold voxel counts, given the fact that this approach strongly depends on the statistical threshold used [18,46]. In the present study, we used a threshold of Z>0. As the ratio of overlap is sensitive to the statistical threshold, altering this threshold to e.g. Z>1.5 would decrease the ratio of overlap (HC: 0.61–0.75 to 0.39–0.59; MS: 0.60–0.75 to 0.38–0.54), whereas the ICC is affected to a much lesser extent (HC: 0.97–0.99 to 0.80–0.99; MS: 0.88–0.99 to 0.80 to 0.96; data not shown). Hence, ICC and overlap ratios produce a different view of reproducibility [47], and both scores attested reasonable to high reproducibility of rfMRI for our sample of patients.

Reproducibility of fMRI activation in patients with MS has been assessed in task-related fMRI, showing high reproducibility of motor function and cognitive function in patients with MS [20,21]. In line with Bosnell et al. (2008), we also found greater variability for patients with MS compared to HC, regarding the ICCs (see also Table 3). For each individual, there are several sources of variability in fMRI (e.g. physiological, such as caffeine ingestion or fatigue; and psychological factors, such as attention and compliance) that can modulate brain response [20,38]. Patients with MS (in the context of widespread cerebral pathology) are certainly likely to bear even more sources of variability. Nevertheless, the fMRI signal in patients is still highly reliable and the ratio of overlap did not differ between HC and patients in our study. Loitfelder et al. (2014) used Bland-Altman plots to explore variability of the fMRI signal changes over time. The percent signal changes noted using this method were stable in HC and patients with MS, indicating a high level of repeatability [21]. Furthermore, patients did not provide more outliers than HC.

In our study, rfMRI networks were highly reproducible not only in HC but also in patients with MS, suggesting that the application of rfMRI to monitor functional changes related to disease progression in MS in clinical practice is reasonable and feasible. Further studies, also accounting for fatigue and anxiety levels of the patients might prove useful to assess the potential influence of behavioral variance on rfMRI.

## Supporting Information

S1 Fig Group functional connectivity maps of all networks. S1 Fig illustrates all nine networks at baseline (BL) and follow-up (FU) for both groups. Tfce corrected p-values > 0.99. HC = Healthy controls, MS = patients with MS.
(TIF)
