# Supplementary figures and images for: Reproducibility of Resting State Connectivity in Patients with Stable Multiple Sclerosis
Source: PLoS One. 2016 Mar 23;11(3):e0152158. doi: 10.1371/journal.pone.0152158 (PMC4805264; doi:10.1371/journal.pone.0152158)

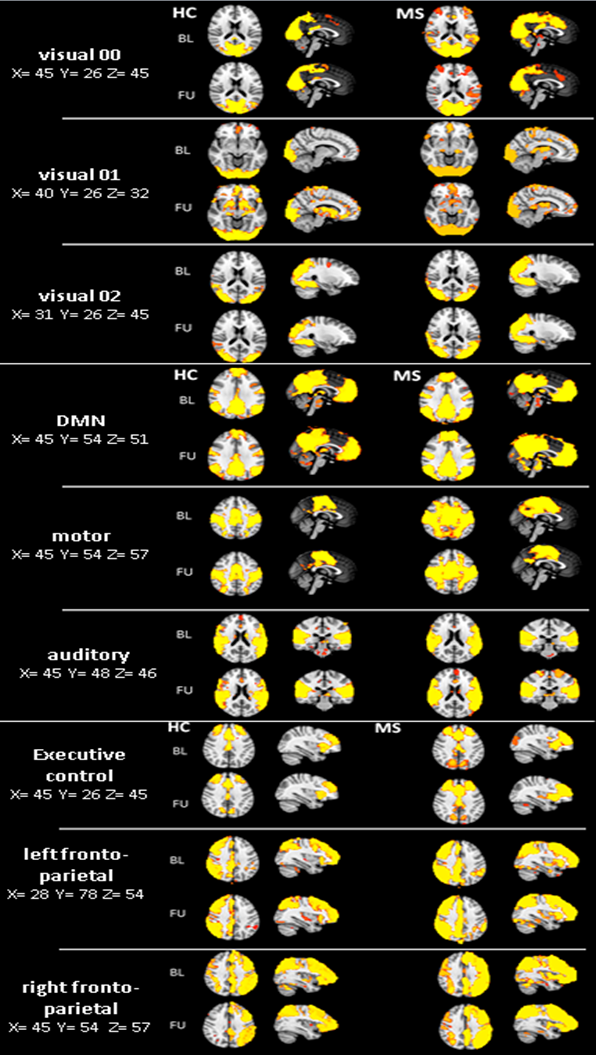

Supplement: S1 Fig — illustrates all nine networks at baseline (BL) and follow-up (FU) for both groups. Tfce corrected p-values > 0.99. HC = Healthy controls, MS = patients with MS. (TIF) [file pone.0152158.s001.tif]
